# Renal plasmacytoma: Report of a rare case and review of the literature

**DOI:** 10.3892/ol.2013.1282

**Published:** 2013-04-03

**Authors:** SHI-QIANG ZHANG, PEI DONG, ZHI-LING ZHANG, SONG WU, SHENG-JIE GUO, KAI YAO, YONG-HONG LI, ZHUO-WEI LIU, HUI HAN, ZI-KE QIN, ZHI-MING CAI, XIAN-XIN LI, FANG-JIAN ZHOU

**Affiliations:** 1 State Key Laboratory of Oncology in Southern China, Department of Urology, Sun Yat-sen University Cancer Center, Guangzhou, Guangdong 510060;; 2 Anhui Medical University, Hefei, Anhui 230022;; 3 Department of Urology, Peking University Shenzhen Hospital, Shenzhen, Guangdong 518036;; 4 Shenzhen Second People’s Hospital, The First Affiliated Hospital of Shenzhen University, Shenzhen, Guangdong 518035, P.R. China

**Keywords:** extramedullary plasmacytoma, kidney, multiple myeloma

## Abstract

Renal plasmacytoma is extremely rare, presenting diagnostic challenges due to its unusual location and non-specific or absent symptoms. To the best of our knowledge, only 24 cases of renal plasmacytoma have been reported in the literature. The present study reports a case of primary renal plasmacytoma in a 46-year-old female patient. Computed tomography (CT) revealed that the mass was located in the lower pole of the left kidney and metastasis was detected in an enlarged para-aortic lymph node. Following careful preparation, a partial nephrectomy was performed and the retroperitoneal lymph node was resected. A pathological examination revealed a renal parenchyma with lymph node involvement; this was confirmed by immunohistochemistry and nested polymerase chain reaction (PCR). Consequently, a diagnosis of a renal extramedullary plasmacytoma (EMP) was proposed. Following this unexpected diagnosis, various examinations were performed, but there was no evidence of systemic plasma cell disease. The patient refused further therapy, including external beam radiotherapy and chemotherapy. Abdominal CT was performed three months post-surgery and did not reveal any relapse. The patient remains disease-free at nine months post-surgery. The current study also presents a review of the literature. Although the general prognosis and outcome of EMP is good, a follow-up examination is recommended due to the possibility of relapse or progression to plasma cell neoplasm (PCN).

## Introduction

Extramedullary plasmacytoma (EMP) is a rare malignant neoplasm that develops due to uncontrolled plasma cell proliferation and monoclonal plasmacytic infiltration (1). The majority of EMPs are detected in the head and neck (2), and the occurrence of an EMP in the kidney is extremely rare. This study presents a case of renal EMP and reviews the existing literature concerning EMPs, as well as multiple myelomas (MMs). EMPs may present as the main symptom of MM, or develop during the course of MM or occasionally occur as solitary tumors. To the best of our knowledge, there are only 24 cases of renal plasmacytoma reported previously (Table I). We report the case of a patient with renal EMP, who may have been diagnosed and treated incorrectly. The study was approved by the Ethics Committee of Sun Yat-sen University Cancer Center, Guangzhou, China. Written informed consent was obtained from the patient.

## Case report

**Figure 1:**
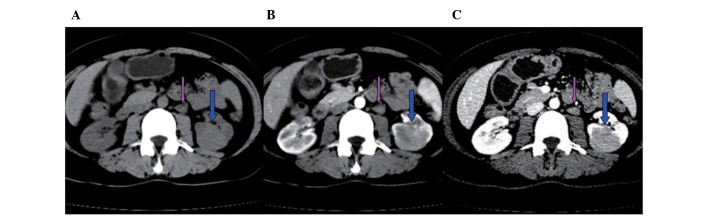
CT scans reveal that the mass (blue arrow) showed enhancement, was located in the lower pole of the left kidney and measured 38×30 mm. Metastasis was detected in an enlarged (20 mm) para-aortic lymph node (pink arrow). CT values; (A) plain scan, 40–60; (B) arterial phase, 71–89; (C) venous phase, 90–120. CT, computed tomography.

A mass in the left kidney was detected in a 46-year-old female patient who underwent ultrasonography as part of a routine physical checkup. Computed tomography (CT) revealed that the mass showed enhancement, was located in the lower pole of the left kidney and measured 38×30 mm. Furthermore, metastasis was detected in an enlarged (20 mm) para-aortic lymph node (Fig. 1). The radiologist suspected a diagnosis of renal cell carcinoma and advised a partial nephrectomy. However, the pre-operative work-up revealed the following: white blood cell (WBC) count, 2.7×10^9^/l; percentage of neutrophils (NE%), 61.8; red blood cell (RBC) count, 3.58×10^12^/l; hemoglobin (HGB) level, 101.1 g/l; platelet (PLT) count, 187.6×10^9^/l; and serum creatinine, calcium and phosphorus levels within normal ranges.

The patient had a 19-year history of hyperthyroidism and an 11-year history of Henoch-Schönlein purpura. The patient had no known drug allergies and their family history was non-contributory. A bone marrow examination was performed next, but it did not reveal any evidence of lymphoma or a plasma cell neoplasm (PCN). The patient was subcutaneously injected with 300 *μ*g recombinant human granulocyte colony-stimulating factor and the complete blood count (CBC) was repeated. The new CBC results were as follows: WBC count, 6.2×10^9^/l; NE%, 84.7; RBC count, 4.01×10^12^/l; HGB level, 110.0 g/l; and PLT count, 133.0×10^9^/l. Subsequently, a partial nephrectomy was performed and the retroperitoneal lymph node was resected.

**Figure 2:**
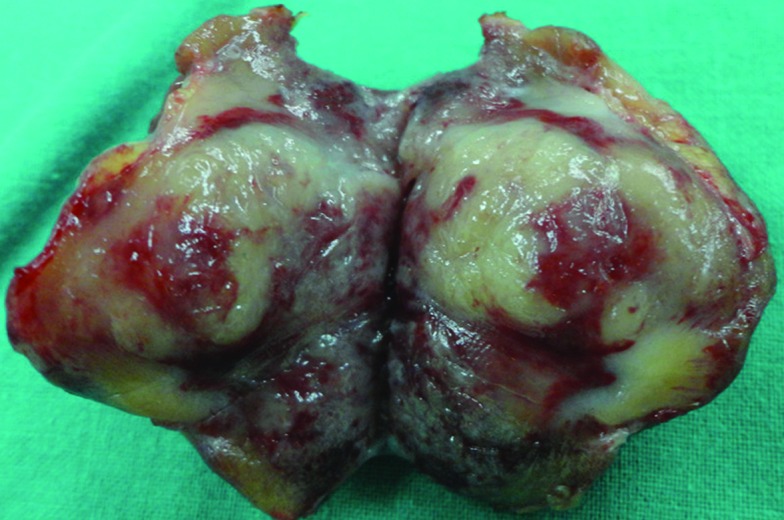
The surgically resected specimen consisted of a segment of renal parenchyma and a 30-mm soft subcapsular mass anchored firmly to the parenchymal section of the kidney, with clear surgical margins. The tumor was a light white and contained hemorrhagic foci.

**Figure 3:**
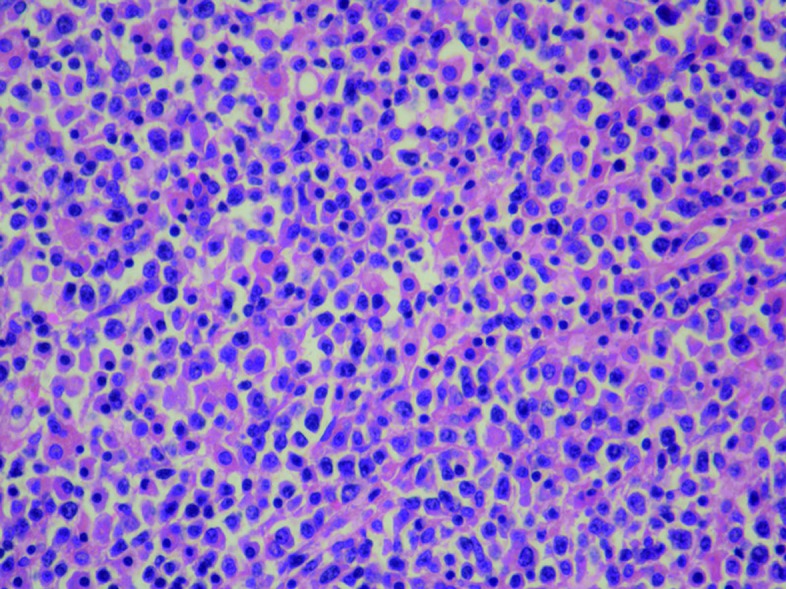
Histological examination showing diffuse infiltration of plasmacytoid cells, which were of different sizes and at various degrees of differentiation. Hematoxylin-eosin staining; magnification, ×400.

The surgically resected specimen consisted of a segment of renal parenchyma and a 30-mm soft subcapsular mass anchored firmly to the parenchymal part of the kidney, with clear surgical margins. The section cut from the tumor was a light white and contained hemorrhagic foci (Fig. 2). Histological examination revealed a 30×25×10 mm unencapsulated mass showing a diffuse infiltration of plasmacytoid cells that were of different sizes and at various degrees of differentiation (Fig. 3). Immunohistochemical analysis demonstrated that the tumor cells were positive for monoclonal κ light chains, CD38, MUM-1, CD138 and VS38C, while certain cells were positive for monoclonal λ light chains. The cells were negative for CD79a, CD5, CD10, L26, UCHL-1, Pax-5, BCL-2, CK and IgD. Ki-67 staining revealed a high cell proliferation rate (>30% immunoreactive cells), indicating the malignant nature of the lesion. Nested polymerase chain reaction (PCR) revealed that the tumor was negative for the rearrangement of the B-cell lymphoma, IgH, and T cell lymphoma, TCRγ, genes. The retroperitoneal lymph node was also involved. Therefore, a diagnosis of a renal EMP was proposed.

Following this unexpected diagnosis, a skeletal survey was performed to complete the tumor staging. The survey did not reveal any lytic bone lesions or evidence of active malignant disease elsewhere. In addition, the urinalysis was positive for Bence-Jones protein. Serum protein electrophoresis excluded MMs and there was no evidence of systemic plasma cell disease.

All these observations were consistent with a diagnosis of an EMP involving the kidney. The patient refused further therapy, including external beam radiotherapy and chemotherapy. Abdominal CT was performed three months post-surgery and did not reveal any relapse. The results of follow-up blood tests were normal and no evidence of hematological disease was noted. The patient remains disease-free at nine months post-surgery.

## Discussion

EMPs may coexist with MM; they may present as the main symptom of MM, develop during the course of MM or occasionally occur as solitary tumors. Laso *et al* considered EMPs and MM to be part of a continuous spectrum of PCNs rather than separate entities (1). The majority of EMPs involve the head and neck region, particularly the upper respiratory tract (2). Others involve diverse anatomical sites, including the gastrointestinal tract, central nervous system, thyroid, breasts, parotid gland, lymph nodes, skin, lungs, pleura, muscle, liver, spleen and pancreas (3,4).

To the best of our knowledge, only 24 cases of renal plasmacytoma have been reported in the literature (Table I) (5–28). However, more cases may have occurred, as this tumor is underdiagnosed and underreported. The clinical suspicion of isolated plasmacytoma is infrequent in patients, with the exception of those with systemic diseases.

Although secondary EMP is much more frequent than primary EMP, almost all cases of renal EMPs registered so far have been primary in nature, that is, without any evidence of an associated PCN.

In cases of renal EMPs, the tumor mass is often confined to this anatomical area and only two cases have shown other organ involvement (7,8). This may reflect the indolent course of renal EMPs.

The diagnosis of an EMP is complex and requires radiological, hematological, biochemical and histological investigation. Primary renal plasmacytomas are not distinguishable from other renal tumors in pre-operative imaging tests. In the present patient, an EMP was diagnosed on the basis of diffuse monoclonal plasma cell infiltration at a single site observed in the immunohistochemical staining for the κ and λ light chains and following the exclusion of a diagnosis of MM (29).

There have been two cases in which an initial diagnosis of renal plasmacytoma was later revised to MM (12,23).

A renal mass in a patient previously diagnosed with EMP is markedly suggestive of tumor recurrence involving the kidney. Among the 24 cases of renal EMPs reported in the literature, five were cases of a renal recurrence of an EMP that had previously occurred at a different site (7,8,10,17,18).

Further tests to rule out MM should include a CBC, serum and urine protein electrophoresis, immunoelectrophoresis, skeletal survey and bone marrow examination (30).

As a result of the lack of typical clinical symptoms and evidence from specific laboratory tests, a diagnosis may be delayed, with potentially disastrous consequences for the patient. In the present study, the patient was initially misdiagnosed with clear cell carcinoma. However, the surgical observations and post-operative pathology confirmed the final diagnosis. Once the diagnosis has been confirmed, the EMP may be staged: stage I, tumors confined to the primary site; stage II, tumors showing local extension or lymph node involvement; and stage III, systemic spread (18,31).

To the best of our knowledge, there are no guidelines for the treatment of renal plasmacytoma. Treatment options for renal plasmacytomas include surgery, chemotherapy and radiotherapy, either alone or in combination (17). Local radiotherapy is the preferred therapeutic modality for EMP owing to its documented radiosensitivity (32). If adjacent nodal involvement is observed, radiation should be applied to these zones as well (33). Kanoh *et al* reported that radiotherapy alone was sufficient to treat their patient (17). In the case of non-renal EMPs, definitive radiation therapy yielded five-year local control rates of >80% and local recurrence rates of <10% (2,34).

Optimal treatment strategies for renal EMPs are difficult to formulate owing to the rarity of the tumors. At present, there is no standard treatment for EMPs involving the kidney, but the current reported experiences of treating primary EMPs indicate that combined therapy (surgery and radiotherapy) is an accepted treatment, depending on the resectability of the lesion. A combination treatment may provide the best results (3).

EMP has a relatively good prognosis, but local recurrence and metastasis develop in 30 and 40% of patients, respectively (35). The five-year survival rate of EMP is excellent at 90%, and previous data suggest that local regression does not necessarily indicate a worse prognosis (31). However, progression to MM does imply this. Three of the reported literature cases of renal plasmacytoma recurred following surgery (23–25), one of which was finally diagnosed as MM.

The periodic evaluation of patients with EMP is necessary due to the possibility of relapse and progression to MM. Physical examinations coupled with laboratory tests, including CBC, renal function tests, analyses of blood calcium, serum albumin and immunoglobulin levels, serum protein electrophoresis, free light chain assays and radiographic studies of the skeleton are required for follow-up.

EMP of the kidney is a rare clinical entity, presenting diagnostic challenges due to its unusual location and nonspecific or absent symptoms. Imaging evaluations may illustrate the existence, size and location of the tumor but are unable to indicate a specific diagnosis. A review of the literature shows that there is currently no widely established standard treatment for EMP of the kidney. If the tumor is located in an area with restricted surgical access, a treatment regimen of local surgery, local radiotherapy or a combination of the two may be initiated. Surgery in combination with radiotherapy may be the best treatment. Although the general prognosis and outcome for EMP is good, a follow-up examination is recommended due to the possibility of relapse or progression to PCN.

## Figures and Tables

**Table I:** Clinical data from 24 cases of renal plasmacytoma.

Authors, year (Ref.)	Age (years)/gender	Tumor location	SPE/IE	Bence-Jones protein	Previously diagnosed PCN	Type of EMP	Clinical manifestations	Treatment	Outcome
Knudsen O, 1937 (5)	46/F	NA	NA	NA	None	P	Palpable mass	Radical nephrectomy	NA
Farrow GM *et al,* 1968 (6)	53/M	L	NA	NA	NA	P	NA	Radical nephrectomy, radiotherapy	Succumbed after 16 years
Solomito VL *et al*, 1972 (7)	64/M	L	NA	NA	Nasopharynx	S	Palpable mass, fatigue	Radical nephrectomy, ileocolectomy	Alive after 4.5 years
Catalona WJ *et al,* 1974 (8)	52/F	R	β ↑	+	Temporal lobe	S	Palpable mass	Biopsy, radiotherapy, chemotherapy	NA
Siemers PT *et al*, 1977 (9)	56/M	L	Normal	NA	None	P	Anorexia, fatigue, palpable mass	Radical nephrectomy, radiotherapy, hemodialysis	Alive after 3 months
Morris SA *et al*, 1977 (10)	50/M	L	IgM ↑	+	Skull, scapula	S	Gross hematuria	Radical nephrectomy, radiotherapy	NA
Silver TM *et al*, 1977 (11)	40/M	L	Normal	-	None	P	Gross hematuria, flank pain	Radical nephrectomy	NA
Kandel LB *et al*, 1984 (12)	55/M	R	γ ↑	+	None	p	Burning feeling,	Radical nephrectomy, radiotherapy	NA
Jaspan T *et al*, 1984 (13)	75/F	L	IgG ↑	+	None	P	Back pain	Biopsy	Succumbed after biopsy
Kanoh T *et al*, 1987 (14)	50/M	R	IgA ↑	NA	MM	S	Microscopic hematuria, abdominal fullness	Chemotherapy, radiotherapy	Succumbed after 2 years
Igel TC *et al*, 1991 (15)	64/M	L	IgM ↑	NA	None	P	Burning feeling, weight loss	Radical nephrectomy, radiotherapy, chemotherapy	NA
Kanoh T *et al*, 1992 (16)	76/F	NA	NA	NA	None	P	NA	Radical nephrectomy	Succumbed after 3 months
Kanoh T *et al*, 1993 (17)	43/M	R	NA	+	Spinal bones	S	Paraplegia	Radiotherapy	NA
Rebelakos AG *et al*, 1995 (18)	52/F	R	NA	NA	T9 vertebra	S	Gross hematuria, back pain, palpable mass	Radical nephrectomy	Alive after 6 months
Shustik C *et al*, 1995 (19)	31/M	NA	IgG ↑	NA	None	P	Asymptomatic	Radical nephrectomy	Alive after 33 months
Manseck A *et al*, 1997 (20)	64/NA	NA	NA	-	None	P	NA	Radical nephrectomy	NA
Tejido Sanchez A *et al,* 2001 (21)	59/NA	R	NA	NA	None	P	NA	Chemotherapy	Succumbed after 1 year
Kim SH *et al*, 2003 (22)	44/F	R	NA	NA	MM	S	Palpable mass	Surgery, chemotherapy, radiotherapy	Alive after 3 months
Fan F *et al*, 2005 (23)	61/F	R	NA	NA	None	P	Back pain	Partial nephrectomy, chemotherapy	Alive after 2.5 year
Park SY *et al*, 2007 (24)	39/M	L	Normal	NA	None	P	NA	Radical nephrectomy	Succumbed after 34 months
Yazici S *et al*, 2009 (25)	67/F	L	α2 ↑, γ ↑	NA	None	P	Asymptomatic	Radical nephrectomy	Alive after 6 months
Mongha R *et al*, 2010 (26)	58/M	R	Normal	-	None	P	Lumbar pain	Radical nephrectomy, radiotherapy	Alive after 1 year
Yang GF *et al*, 2010 (27)	76/F	L	NA	NA	None	P	Back pain	Radical nephrectomy	NA
Zhong Y *et al*, 2010 (28)	41/M	L	NA	-	None	P	Epigastric discomfort	Radical nephrectomy	NA
Present case	46/F	L	γ ↑, α1 ↑	+	None	P	NA	Radical nephrectomy	Alive after 6 months

F, female; M, male; L, left kidney; R, right kidney; NA, not available; SPE, serum protein electrophoresis; IE, immunoelectrophoresis;

↑, increased;

+, positive;

−, negative; PCN, plasma cell neoplasm; MM, multiple myeloma; EMP, extramedullary plasmacytoma; P, primary; S, secondary.
